# DHAV-1 Blocks the Signaling Pathway Upstream of Type I Interferon by Inhibiting the Interferon Regulatory Factor 7 Protein

**DOI:** 10.3389/fmicb.2021.700434

**Published:** 2021-11-12

**Authors:** Yalan Lai, Xiaoyan Xia, Anchun Cheng, Mingshu Wang, Xumin Ou, Sai Mao, Di Sun, Shaqiu Zhang, Qiao Yang, Ying Wu, Dekang Zhu, Renyong Jia, Shun Chen, Mafeng Liu, Xin-Xin Zhao, Juan Huang, Qun Gao, Bin Tian, Yunya Liu, Yanling Yu, Ling Zhang, Leichang Pan

**Affiliations:** ^1^Institute of Preventive Veterinary Medicine, Sichuan Agricultural University, Chengdu, China; ^2^Key Laboratory of Animal Disease and Human Health of Sichuan Province, Sichuan Agricultural University, Chengdu, China; ^3^Avian Disease Research Center, College of Veterinary Medicine, Sichuan Agricultural University, Chengdu, China

**Keywords:** duck hepatitis A virus type I, 3C protein, interferon regulatory factor 7, immune regulation, type I interferon

## Abstract

Duck hepatitis A virus (DHAV), which mainly infects 1- to 4-week-old ducklings, has a fatality rate of 95% and poses a huge economic threat to the duck industry. However, the mechanism by which DHAV-1 regulates the immune response of host cells is rarely reported. This study examined whether DHAV-1 contains a viral protein that can regulate the innate immunity of host cells and its specific regulatory mechanism, further exploring the mechanism by which DHAV-1 resists the host immune response. In the study, the dual-luciferase reporter gene system was used to screen the viral protein that regulates the host innate immunity and the target of this viral protein. The results indicate that the DHAV-1 3C protein inhibits the pathway upstream of interferon (IFN)-β by targeting the interferon regulatory factor 7 (IRF7) protein. In addition, we found that the 3C protein inhibits the nuclear translocation of the IRF7 protein. Further experiments showed that the 3C protein interacts with the IRF7 protein through its N-terminus and that the 3C protein degrades the IRF7 protein in a caspase 3-dependent manner, thereby inhibiting the IFN-β-mediated antiviral response to promote the replication of DHAV-1. The results of this study are expected to serve as a reference for elucidating the mechanisms of DHAV-1 infection and pathogenicity.

## Introduction

Duck hepatitis A virus (DHAV), which belongs to genus *Avihepatovirus* in the *Picornaviridae* family, mainly infects 1- to 4-week-old ducklings with a fatality rate of 95% and poses a huge economic threat to the duck industry. Clinical observation has shown that the infected ducklings show neurological symptoms such as opisthotonos, spasm, and convulsion. In addition, the livers of the ducklings are enlarged, and hemorrhagic spots are observed after the anatomical examination (Ou et al., 2017). DHAV is divided into three serotypes [DHAV-1, DHAV-2 (Tseng and Tsai, 2007), and DHAV-3 (Kim et al., 2007; Wen et al., 2014)], and the main most common serotypes in China are DHAV-1 and DHAV-3 (Wen et al., 2018). The DHAV-1 genome is a single-stranded, positive-sense RNA with only one open reading frame (ORF) that encodes one polyprotein. This polyprotein is then cleaved into the structural proteins VP0, VP3, and VP1 and the non-structural proteins 2A1, 2A2, 2A3, 2B, 2C, 3A, 3B, 3C, and 3D under the cleavage activity of viral cysteine protease (Sun et al., 2017; Yang et al., 2017; Kloc et al., 2018).

To resist invasion from various viruses, host cells have established complex antiviral defense systems against viral infections, among which the innate immune response is the first line of defense against viral infection. Retinoic acid-induced gene 1 (RIG-1) and melanoma differentiation-related gene 5 (MDA-5) are the main receptors for the recognition of RNA viruses. They quickly recognize double-stranded RNA (dsRNA) (Lei et al., 2016), the replicative intermediate of the virus, and recruit the mitochondrial antiviral signal (MAVS, also known as IPS-1 and VISA) protein (Kawai et al., 2005). The subsequent recruitment of nuclear factor kappa-B kinase epsilon (Iκκε) and TANK binding kinase 1 (TBK1) results in the phosphorylation and dimerization of interferon (IFN) regulatory factor 3 (IRF3) or IFN regulatory factor 7 (IRF7) (Han et al., 2004; Croft et al., 2017). Finally, dimerized IRF3 or dimerized IRF7 enters the nucleus and initiates the transcription of type I IFN.

Picornaviruses have evolved many methods to resist the antiviral mechanisms of host cells to break through the innate immune defense and effectively replicate in host cells. For example, RIG-I was found to be cleaved in cells infected with poliovirus (PV) (Barral et al., 2009), and subsequent studies found that the 3C protein of PV directly cleaves RIG-I through its proteolytic enzyme activity (Papon et al., 2009). Coxsackievirus B3 (CVB3) also uses the same mechanism to inhibit RIG-I (Feng et al., 2014). In addition, MAVS can be directly cleaved through the 2A proteins of these viruses (Feng et al., 2014). Enterovirus 71 (EV71) inhibits the formation of a complex between RIG-1 and MAVS by interacting with the caspase-recruitment domain at the N-terminus of RIG-I, thereby blocking the recruitment of MAVS and subsequent nuclear translocation of IRF3 (Lei et al., 2010). In addition, EV71 blocks the host cell immune response by lysing IRF7 and MAVS, ultimately promoting the replication and proliferation of the virus (Lei et al., 2013; Wang et al., 2013). Coxsackievirus A16 (CV-A16), coxsackievirus A6 (CV-A6), and enterovirus D68 (EV-D68) inhibit the interaction between MDA5 and MAVS by binding MDA5 (Rui et al., 2017). Encephalomyocarditis virus (EMCV) disrupts the formation of the TANK–TBK1–IKKε–IRF3 complex by lysing the scaffold protein TANK, which is required by the TBK1–IKKε complex, thereby inhibiting IRF3 phosphorylation and reducing the production of type I IFN (Huang et al., 2017). The 2B protein and the intermediate 3ABC protein of hepatitis A virus (HAV) can interact with MAVS, thereby interfering with the formation of the Iκκε–TBK1 complex and ultimately blocked IRF3 activation (Paulmann et al., 2008).

In summary, enterovirus-mediated interference with the type I IFN pathway in infected cells is a common immune evasion strategy. However, unlike other picornaviruses, the mechanism of immune regulation by DHAV-1 is rarely reported. Therefore, determining whether DHAV-1 regulates the immune response of host cells and the specific mechanism by which it regulates immunity is of great importance for antiviral research and clinical applications.

## Materials and Methods

### Strain and Antibody

The DHAV-1 H strain (GenBank accession number: JQ301467.1), the engineered *E. coli* DH5α bacterium, HEK293T human embryonic kidney cells, and duck embryo fibroblasts (DEFs) used in this study were all preserved and provided by the Sichuan Agricultural University Poultry Disease Prevention Research Center. A mouse anti-Flag monoclonal antibody (Cat: M185-3S) and a mouse anti-HA monoclonal antibody (Cat: M132-3) were purchased from Medical & Biological Laboratories Co., Ltd. A rabbit anti-duck IRF7 polyclonal antibody, a HRP-conjugated goat anti-mouse IgG heavy chain antibody (Cat: AS064), a HRP-conjugated goat anti-mouse IgG light chain antibody (Cat: AS062) and a mouse anti-GFP antibody (Cat: AE012) were prepared by ABclonal Technology Co., Ltd. A rabbit anti-beta (β)-actin antibody (Cat: 20536-1-AP) was obtained from Proteintech Co., Ltd. A rabbit anti-HA monoclonal antibody (Cat: AF2305), a mouse IgG antibody (Cat: A7028), a HRP-conjugated goat anti-mouse IgG (Cat: A0216) and a HRP-conjugated goat anti-rabbit IgG (Cat: A0208) were purchased from Beyotime Co., Ltd. A rabbit anti-Histone H3 (Cat: TA6358) and a mouse anti-beta (β)-tubulin monoclonal antibody (Cat: T63017-2) were purchased from Abmart Co., Ltd. A rabbit anti-VP3 antibody was prepared in our laboratory (Shen et al., 2016).

### Plasmids

pCMV-3C-HA, pCMV-3C_*H*__38__*A*_-HA, pCAGGS, pCAGGS-MDA5-Flag, pCAGGS-RIG-I(N-terminal)-Flag, pCAGGS- MAVS-Flag, pCAGGS-TBK1-Flag, pCAGGS-IRF7-Flag, IFN-β-Luc (Chen et al., 2019), pRSL-TK, and the eukaryotic expression vector pCMV were preserved and provided by the Sichuan Agricultural University Poultry Disease Prevention Research Center. shRNA-NC and pGPU6/GFP/Neo-IRF7-129/399 (renamed shRNA-IRF7-1/2) were designed and synthesized by Shanghai Gene Pharma Co., Ltd. The sequence of shRNA-IRF7-1 is 5′-GGAGACCTCCATCTTCGACTT-3′. The sequence of shRNA-IRF7-2 is 5′-GCTCATCGAGCAGTACAACAT-3′. The primers used in the experiment were synthesized by Sangon Biotech (Shanghai) Co., Ltd.; their sequences are shown in Table 1.

**TABLE 1:** The sequences of all primers used in this study.

Gene	Primer name	Primer sequence
IFN-β	P1 F	CCTCAACCAGATCCAGCATT
	P1 R	GGATGAGGCTGTGAGAGGAG
β-Actin	P4 F	TACGCCAACACGGTGCTG
	P4 R	GATTCATCATACTCCTGCTTG
Mx	P2 F	TGCTGTCCTTCATGACTTCG
	P2 R	GCTTTGCTGAGCCGATTAAC
OASL	P3 F	TCTTCCTCAGCTGCTTCTCC
	P3 R	ACTTCGATGGACTCGCTGTT
pEGFP-C1-3C-P1	P5 F	TACAAGTCCGGACTCAGATCTAGC GGGCGGGTGAATTTCAGACATA
	P5 R	GTACCGTCGACTGCAGAATTCTTAG GGTTCTCCATCATAAGTAATGGGG
pEGFP-C1-3C-P2	P6 F	TACAAGTCCGGACTCAGATCTAAG GGGACCCCCATTACTTATGATG
	P6 R	GTACCGTCGACTGCAGAATTCTTAA GTTGTTGTGGAACCAAAAGGCCTA
pEGFP-C1-3C-P3	P7 F	TACAAGTCCGGACTCAGATCTACA AAGATTAGGCCTTTTGGTTCCA
	P7 R	GTACCGTCGACTGCAGAATTCTTAT TGATTAAAAACTGGAAAGACCCTA

### Plasmid Preparation and Transfection

Endotoxin-free plasmid was extracted from the cloned bacterial liquid containing the target plasmid using Endo-Free Plasmid DNA Mini Kit (Omega) according to the instructions, and the plasmid DNA was stored at −20°C.

Duck embryo fibroblast cells were prepared and cultured in 6-well culture dishes and 12-well culture dishes. The transfection of six-well cell culture dishes was carried out according to the instructions of the Lipofectamine^®^ 2000 Reagent (Invitrogen). Taking one well as an example, the mixture of Opti-MEM medium and 8 μL transfection reagent was incubated at room temperature for 5 min, and then incubated with 4 μg plasmid at room temperature for 20 min to form a complex. Finally, this mixture was added to a six-well cell culture plate. The transfection of 12-well cell culture dishes was carried out according to the instructions of the TransIntro^TM^ EL Transfection Reagent (TransGen Biotech, Beijing, China). Taking one well as an example, 1.6 μg plasmid was diluted in Opti-MEM medium, and then 3.2 μL transfection reagent was added to mix, incubated at room temperature for 15 min, and the mixture was added to a cell culture dish.

### Dual-Luciferase Reporter Gene System

For this experiment, we referred to the manual of the Dual-Luciferase^®^ Reporter (DLR^TM^) Assay System (Promega). Briefly, samples were collected 36 h after transfection. We discarded the culture medium, diluted the 1× cell lysate, and stored it at −80°C. Subsequently, we added 75 μL of the sample and 75 μL of Dual-Glo^®^ luciferase detection reagent to a 96-well plate and detected the fluorescence signal to obtain the value A. Finally, we added 75 μL of Stop & Glo^®^ detection reagent and detected the fluorescence signal to obtain the value B. The value of A was divided by the value of B to obtain the final result.

### qRT-PCR

Total RNA was isolated from the samples using RNAiso Plus reagent (TaKaRa) according to the instructions. Viral copy number was determined by One-Step TaqMan real-time fluorescent quantitative RT-PCR (Miao et al., 2008; Hu et al., 2016).

### Western Blot Analysis

The supernatant of the cells was discarded 36 h after transfection, and the cells were suspended in RIPA lysis buffer (strong) (Biyuntian). The resulting proteins were separated by 12% SDS-PAGE and then transferred to a PVDF membrane. The membrane was blocked with 5% skimmed milk powder at 37°C for 3 h and incubated overnight (4°C) with mouse anti-Flag (1:4000), mouse anti-HA (1:10,000), and rabbit anti-VP3 (1:800) primary antibodies. HRP-labeled goat anti-rabbit IgG (1:3000) or HRP-labeled goat anti-mouse IgG (1:3000) was used as the secondary antibody and incubated with the blot for 1 h at 37°C.

### Coimmunoprecipitation

After 36 h of transfection, the cells were lysed with NP40 lysis buffer (Shanghai Biyuntian Biotechnology Co., Ltd.). We collected the lysate supernatant and divided it into two samples. Mouse anti-IgG (control group) and mouse anti-Flag monoclonal antibodies were added to the two samples at a ratio of 1:100 and incubated at 4°C for 24 h. Then, we added protein A and protein G to the samples at a ratio of 1:10 and incubated them at 4°C for 12 h. Finally, we discarded the supernatants of all samples and added 40 μL of PBS and 10 μL of 5× loading buffer. The subsequent steps were the same as those used for western blotting.

### Nuclear and Cytoplasmic Separation

We transfected DEFs with pCMV-3C-HA, pCAGGS-IRF7-Flag, pCMV-HA, and poly(I:C) according to the appropriate experimental group and used NP40 lysis buffer (Shanghai Biyuntian Biotechnology Co., Ltd.) to collect cell samples 36 h after transfection. The samples were placed on ice for 30 min and centrifuged at 12,000 rpm for 10 min. The supernatants were aspirated into new EP tubes, the pellets were washed three times with PBS, and 1% SDS lysis buffer was added to lyse the cells on ice for 30 min, after which western blot detection was performed.

### Construction of pEGFP-C1-3C-P1/P2/P3 Eukaryotic Plasmids

We designed upstream and downstream primers to specifically amplify a truncated fragment of 3C (Table 1). Subsequently, we used these primers and PrimeSTAR Max (TaKaRa) for specific amplification of the fragment and recovered the amplified products. The vector pEGFP-C1 was linearized using the restriction sites for which the primers were designed (*Bgl*II and EcoRI), after which the digested products were recovered, and MonClone^TM^ Single Assembly Cloning Mix (Monad) was used for homologous recombination. Positive colonies were identified by PCR using 2× Taq Master Mix (Vazyme) (Lai et al., 2019). Positive clones were sequenced by Sangon Biotech (Shanghai) Co., Ltd.

### Treatment of Cells With Inhibitors of Protein Degradation Pathways

We transfected DEFs with pCMV-3C-HA. After 24 h of transfection, the inhibitors Z-VAD-FMA (50 μM), MG-132 (20 μM), NH_4_Cl (10 mM), Z-DEVD-FMK (20 μM), Z-IETD-FMK (30 μM), and Z-LEHD-FMK (20 μM) were individually added to the cell culture medium. Cell samples were collected after 12 h of treatment with the inhibitors and analyzed by western blotting.

### shRNA-Mediated IRF7 Knockdown

When the cell density reached 70–90%, transfection began. We transfected the DEFs with shRNA-IRF7-1 and shRNA-IRF7-2, and another group of cells was transfected with shRNA-NC as a control group. The cells were inoculated with the DHAV-1 CH strain (MOI = 0.4) 24 h after transfection. Cell samples were collected 36 h after transfection.

## Results

### The 3C, 3A, and 3AB Proteins Inhibited the Poly(I:C)-Induced Signaling Pathway Upstream of Interferon-β

**FIGURE 1:**
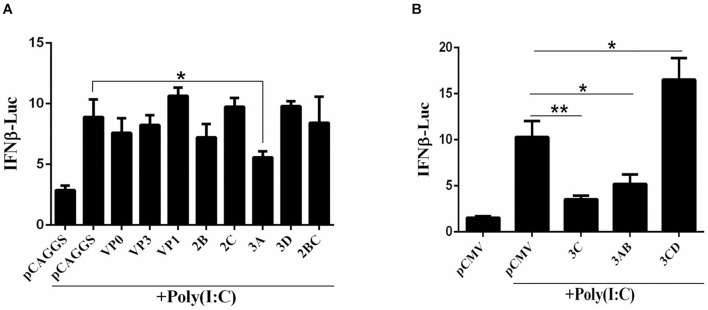
**(A,B)** The effect of DHAV-1 viral proteins on the IFN-β signaling pathway induced by poly(I:C). pCAGGS, pCAGGS-VP1-Flag, pCAGGS-VP0-Flag, pCAGGS-VP3-Flag, pCAGGS-2B-HA, pCAGGS-2C-HA, pCAGGS-3A-Flag, pCAGGS-3D-Flag, pCAGGS-2BC-HA, pCMV, pCMV-3C-HA, pCMV-3CD-HA, and pCMV-3CD-HA were cotransfected into DEFs with IFN-β pro-Luc, poly(I:C), and pRL-TK. The transfection ratio of other plasmids was 1:1:1, except for the reference plasmid, pRL-TK. The amount of pRL-TK used for transfection was 1/20 that of IFN-β pro-Luc. Cell samples were collected 36 h after transfection, and IFN-β promoter activity was detected using a dual-luciferase reporter system. *P < 0.05, **P < 0.01, compared with control group.

The DHAV-1 genome is a single-stranded, positive-sense RNA that forms dsRNA replication intermediates during the replication process. To explore the influence of each viral protein on the pathway upstream of IFN-β that recognizes dsRNA, the dsRNA mimic poly(I:C) was used to mimic the dsRNA formed during RNA viral infection, and the effect of the viral proteins on IFN-β promoter activation was investigated by using the dual-luciferase reporter gene system. The 3C, 3A, and 3AB proteins significantly inhibited activation of the IFN-β promoter induced by poly(I:C) (Figure 1).

### The 3C Protein Inhibited the Poly(I:C)-Induced Interferon-β Signaling Pathway Independent of Its Protease Activity

**FIGURE 2:**
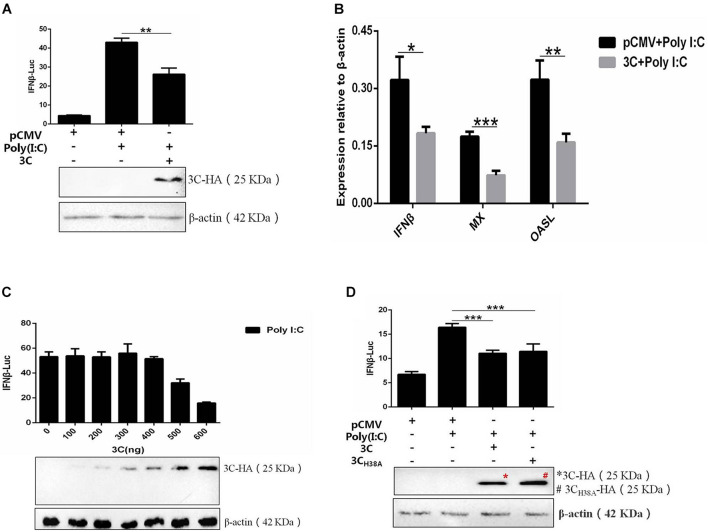
The DHAV-1 3C protein inhibits the poly(I:C)-induced IFN-β signaling pathway. **(A)** pCMV-3C-HA, poly(I:C), IFN-β pro-Luc, and the reference plasmid (pRL-TK) were cotransfected into DEFs (except for the reference plasmid, pRL-TK, the transfection ratio was 3:1:1, and the amount of pRL-TK used for transfection was 1/20 that of IFN-β pro-Luc). Cell samples were collected 36 h after transfection, and IFN-β promoter activity was detected using a dual-luciferase reporting system to explore the effect of the 3C protein on activation of the IFN-β promoter induced by poly(I:C). **(B)** pCMV-3C-HA and poly(I:C) were cotransfected into DEFs (transfection ratio of 1:1). Cell samples were collected 36 h after transfection, and the total RNA was extracted from the samples. Quantitative PCR analysis of the effect of the 3C protein on poly(I:C)-induced IFN-β and IFN-β-induced MX and OASL mRNA levels. **(C)** A total of 0.3 μg of poly(I:C), 0.3 μg of IFN-β pro-Luc, the internal reference plasmid pRL-TK, and different amounts of pCMV-3C-HA were cotransfected into DEFs (the amount of pRL-TK transfected was 1/20 that of IFN-β pro-Luc). Thirty-six hours after transfection, cell samples were collected, and the dual-luciferase reporter system was used to detect IFN-β promoter activity to explore the effects of differences in 3C protein expression on IFN-β promoter activation. **(D)** pCMV-3C_*H*__38__*A*_-HA, poly(I:C), IFN-β pro-Luc, and the reference plasmid pRL-TK were cotransfected into DEFs (except the reference plasmid pRL-TK, the transfection ratio was 3:1:1, and the amount of pRL-TK transfected was 1/20 that of IFN-β pro-Luc). Thirty-six hours after transfection, cell samples were collected. The dual-luciferase reporter system was used to detect IFN-β promoter activity to explore the loss of 3C protease activity on IFN-β promoter activation induced by poly(I:C). *P < 0.05, **P < 0.01, ***P < 0.001, compared with the control group.

The 3C proteins of many picornaviruses can inhibit the production of type I IFN by cleaving the receptors, adaptor proteins, and regulators involved in the signal transduction pathway, but the 3C protein of DHAV-1 has not been reported. Therefore, we selected the 3C protein as the research object for this study and once again explored its influence on the signaling pathway upstream of IFN-β. The 3C protein significantly inhibited IFN-β promoter activation induced by poly(I:C) (Figure 2A) and also significantly inhibited the upregulation of IFN-β and IFN-β-induced increases in MX and OASL mRNA levels (Figure 2B). Subsequently, poly(I:C) was cotransfected with pCMV-3C-HA at different amounts to explore the effect of different 3C protein expression levels on IFN-β promoter activity. The inhibitory effect of the 3C protein on poly(I:C)-induced IFN-β promoter activation gradually increased with increasing 3C protein expression (Figure 2C). The 3C protein of DHAV-1 has proteolytic enzyme activity, and this feature is of great importance for its various functions. When the histidine 38 in the 3C protein was mutated to alanine, the 3C protein lost its proteolytic enzyme activity (Sun et al., 2017). We cotransfected the mutant plasmid pCMV-3C_*H*__38__*A*_-HA and poly(I:C) to explore whether inhibition of the pathway upstream of IFN-β by the 3C protein depends on its proteolytic enzyme activity. The results showed that the 3C protein could still inhibit activating the IFN-β promoter induced by poly(I:C) after its proteolytic activity was lost (Figure 2D).

### The 3C Protein Inhibits Interferon-β Induction Through the Interferon Regulatory Factor 7 Protein

**FIGURE 3:**
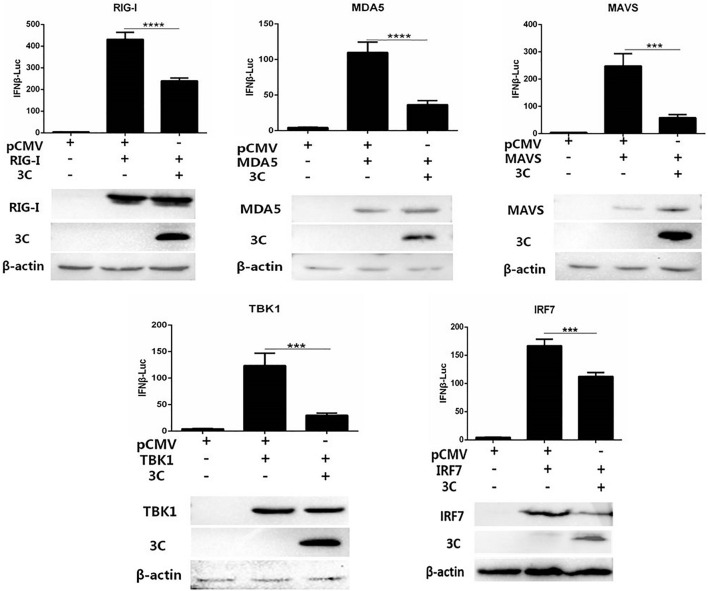
Through the IRF7 protein, the 3C protein inhibits the IFN-β signaling pathway. pCAGGS-MDA5-Flag, pCAGGS-RIG-I(N-terminal)-Flag, pCAGGS-MAVS-Flag, pCAGGS-TBK1-Flag, and pCAGGS-IRF7-Flag were combined with pCMV-3C-HA and IFN-β pro-Luc, and the reference plasmid pRL-TK and cotransfected into DEFs (the transfection ratio of the first three plasmids was 1:3:1, and the amount of pRL-TK transfected was 1/20 that of IFN-β pro-Luc). Six hours after transfection, cell samples were collected. The dual-luciferase reporter system was used to detect IFN-β promoter activity and screen target molecules through which the 3C protein inhibits the pathway upstream of IFN-β. ***P < 0.001, ****P < 0.0001, compared with control group.

We combined pCAGGS-MDA5-Flag, pCAGGS-RIG-I(N-terminal)-Flag, pCAGGS-MAVS-Flag, pCAGGS-TBK1-Flag, pCAGGS-IRF7-Flag, and pCMV-3C-HA and then cotransfected them with IFN-β-Luc and RL-TK into DEFs to explore the target by which the 3C protein inhibits the IFN-β signaling pathway. The 3C protein significantly inhibited activation of the IFN-β promoter induced by MDA5, RIG-I (N-terminal), MAVS, TBK1, and IRF7 (Figure 3), indicating that the target of the 3C protein by which it inhibits the pathway upstream of IFN-β is the IRF7 protein.

### The 3C Protein Inhibits Nuclear Translocation of the Interferon Regulatory Factor 7 Protein

**FIGURE 4:**
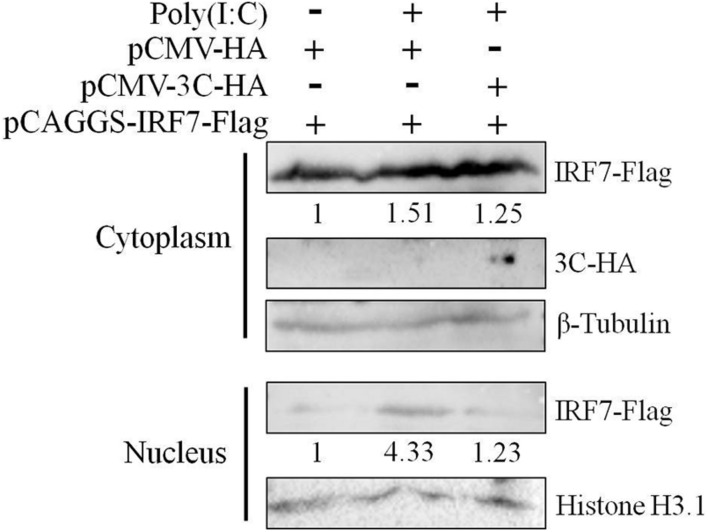
Effect of the 3C protein on nuclear translocation of the IRF7 protein. HEK293T cells were divided into three experimental groups. The first group was transfected with 0.5 μg of pCAGGS-IRF7-Flag and 0.5 μg of pCMV-HA, the second group was transfected with 0.5 μg of pCAGGS-IRF7-Flag, 0.5 μg of pCMV-HA, and 1 μg of poly(I:C); and the third group was transfected with 0.5 μg of pCAGGS-IRF7-Flag, 0.5 μg of pCMV-3C-HA and 1 μg of poly(I:C). Cell samples were collected for nuclear and cytoplasmic separation at 36 h after transfection. Tubulin and histone H3.1 were used as loading controls for the cytosolic and nuclear fractions, respectively.

Since nuclear translocation of the activated IRF7 protein is critical to its transcriptional activation function, we first explored the effect of the 3C protein on the nuclear translocation of the IRF7 protein. We transfected DEFs with pCAGGS-IRF7-Flag alone or cotransfected DEFs with pCMV-3C-HA and poly(I:C). Compared with that, when the IRF7 plasmid was transfected alone, the nuclear and cytoplasmic expression of the IRF7 protein was increased when poly(I:C) was used as a stimulus. When the 3C and IRF7 proteins were coexpressed, the nuclear protein expression of IRF7 was still reduced even when poly(I:C) was used as a stimulus (Figure 4).

### The 3C Protein Reduces the Protein Expression of Interferon Regulatory Factor 7 Independent of Its Proteolytic Enzyme Activity

**FIGURE 5:**
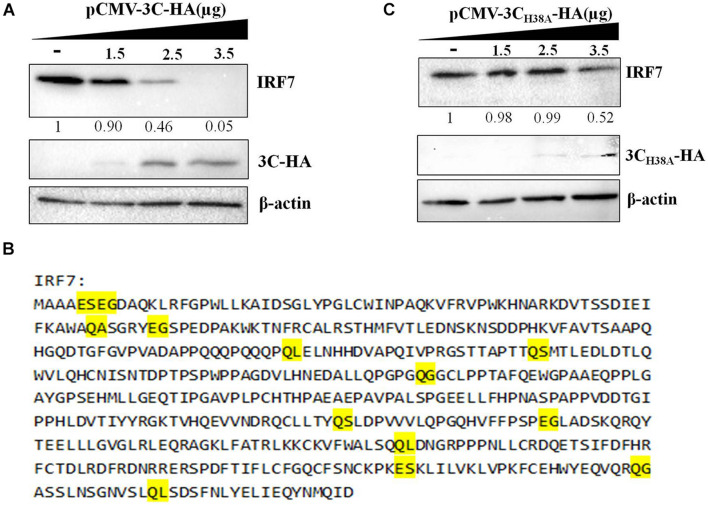
Effect of the 3C protein on the IRF7 protein level. pCAGGS-IRF7-Flag (0.5 μg) was cotransfected with different pCMV-3C-HA or pCMV-3C_*H*__38__*A*_-HA (the amount transfected into each group was 4 μg, and pCMV-HA was used to supplement the insufficient group). Cell samples were collected at 36 h after transfection to detect the protein expression of IRF7. **(A)** Effect of the 3C protein on IRF7 protein expression in DEFs. **(B)** The possible cleavage site for the 3C protein in the amino acid sequence of the IRF7 protein. **(C)** Effect of the 3C protein lacking proteolytic enzyme activity on the protein expression of IRF7.

The 3C protein of EV71 can directly cleave IRF7 into two fragments that cannot activate the expression of type I IFN (Lei et al., 2013). Since the DHAV-1 3C protein has proteolytic enzyme activity, we explored whether it could cleave the IRF7 protein. pCAGGS-IRF7-Flag was transfected into DEFs, and the protein expression of IRF7 was detected by western blotting. We did not detect the band corresponding to IRF7 after IRF7 cleavage. However, as the protein expression of 3C increased, the protein expression of IRF7 gradually decreased (Figure 5A). The 3C protein of picornavirus acts on many cleavage sites, including Q-G/N/S/A/L and E-G/S (Wang et al., 2014; Di et al., 2016). We further analyzed possible 3C cleavage sites in the IRF7 protein and identified many possible 3C cleavage sites in IRF7 (Figure 5B). Therefore, we speculate that the 3C protein cleaves the IRF7 protein into small protein fragments that cannot be detected by western blotting. To verify this hypothesis, we transfected pCMV-3C_*H*__38__*A*_-HA into DEFs to explore the correlation between decreased IRF7 protein expression and the proteolytic enzyme activity of the 3C protein. The results showed that after the 3C protein lost its proteolytic enzyme activity, the IRF7 protein level gradually decreased with increasing 3C protein expression (Figure 5C).

### The 3C Protein Interacts With the Interferon Regulatory Factor 7 Protein Through Its N-Terminus

**FIGURE 6:**
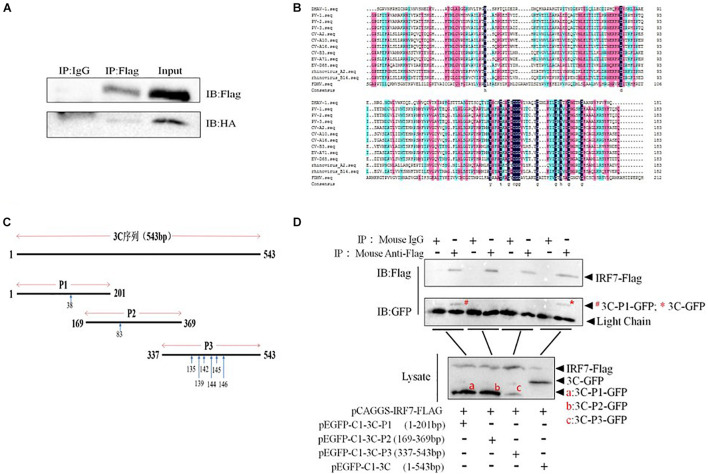
Interaction between the 3C and IRF7 proteins. HEK-293T cells were transfected with pCAGGS-IRF7-Flag and pCMV-3C-HA, pEGFP-C1-3C-P1, pEGFP-C1-3C-P2, pEGFP-C1-3C-P3, or pEGFP-C1-3C at a transfection ratio of 1:3, and 36 h after transfection, cell samples were collected for immunoprecipitation experiments. **(A)** The interaction between the 3C and IRF7 proteins. **(B)** Comparison of 3C protein amino acid sequences. **(C)** Schematic diagram of the truncated 3C protein plasmid, with blue arrows indicating conserved amino acid positions. **(D)** The region through which the 3C protein binds the IRF7 protein was screened.

Then, we explored whether the 3C protein and IRF7 protein interact. The results showed that when IRF7-Flag was used to pull down proteins, bands containing the 3C protein were detected, indicating that the 3C protein can interact with the IRF7 protein (Figure 6A). The amino acid sequence of the DHAV-1 3C protein was compared with the 3C protein sequences of other picornaviruses. The results showed that amino acids 38, 83, 135, 139, 142, 144, 145, and 146 of the DHAV-1 3C protein are highly conserved (Figure 6B). We truncated the 3C protein into 3 segments (P1/P2/P3) based on the conserved sites in the 3C protein. The P1 segment contained amino acid 38, the P2 segment contained amino acid 83, and the P3 segment contained 135, 139, 142, 144, 145, and 146 (Figure 6C). Subsequently, coimmunoprecipitation was performed again to explore where the 3C protein interacts with the IRF7 protein. The results showed that when IRF7-Flag was used to pull down proteins, only protein bands containing 3C-P1-GFP and 3C-GFP were detected (Figure 6D), indicating that the 3C protein interacts with the IRF7 protein through its N-terminus.

### The 3C Protein Reduces the Protein Level of Interferon Regulatory Factor 7 in a Caspase 3-Dependent Manner

**FIGURE 7:**
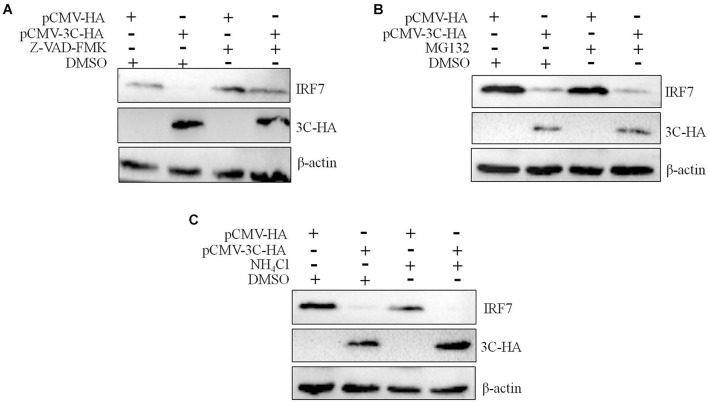
The degradation of IRF7 induced by the 3C protein is caspase-dependent. A total of 1.6 μg of pCMV-3C-HA and 1.6 μg of pCMV-HA were transfected into DEFs. Twenty-four hours after transfection, the broad-spectrum caspase inhibitor Z-VAD-FMA (50 μM), proteasome inhibitor MG132 (20 μM), autophagy inhibitor 3-MA (10 mM), and autophagosome-lysosome inhibitor NH_4_Cl (10 mM) were added to the cell culture medium, with a DMSO negative control group set up at the same time. After 12 h of treatment, cell samples were collected and subjected to immunoblotting experiments to detect the endogenous IRF7 expression level in the presence or absence of the inhibitors. **(A)** The effect of Z-VAD-FMK on IRF7 protein degradation. **(B)** The effect of MG132 on IRF7 protein degradation. **(C)** The effect of NH_4_Cl on IRF7 protein degradation.

**FIGURE 8:**
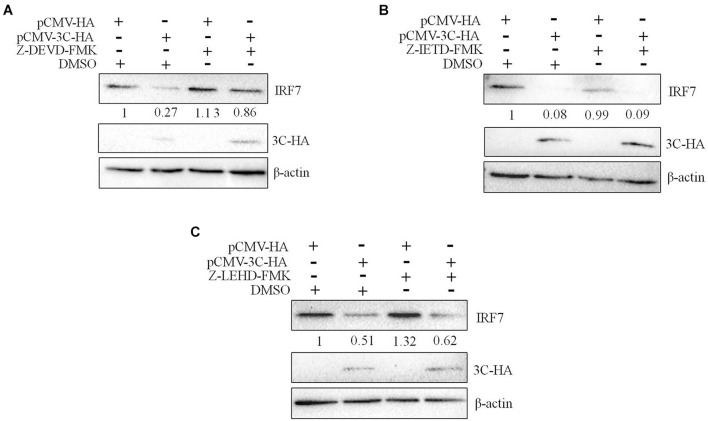
The degradation of IRF7 induced by the 3C protein depends on caspase 3. A total of 1.6 μg of pCMV-3C-HA and 1.6 μg of pCMV-HA were transfected into DEFs. Twenty-four hours after transfection, the caspase 3 inhibitor Z-DEVD-FMK (20 μM), caspase 8 inhibitor Z-IETD-FMK (30 μM), and caspase 9 inhibitor Z-LEHD-FMK (20 μM) were added to the cell culture medium. After 12 h of treatment, cell samples were collected and subjected to immunoblotting experiments to detect the endogenous IRF7 expression level in the presence or absence of the inhibitors. **(A)** The effect of Z-DEVD-FMK on IRF7 degradation. **(B)** The effect of Z-IETD-FMK on IRF7 degradation. **(C)** The effect of Z-LEHD-FMK on IRF7 degradation.

We had proven that 3C protein affects the protein level of IRF7 independent of its proteolytic enzyme activity. Therefore, we speculated that the 3C protein indirectly decreases the protein expression of IRF7 in DEFs through the protein degradation pathway. In eukaryotic cells, the main protein degradation pathways are the lysosomal pathway, proteasome pathway, and caspase-dependent pathway (Wang et al., 2017). To determine which protein degradation pathway is involved in the 3C-induced reduction in IRF7 protein expression, we treated the cells with the broad-spectrum caspase inhibitor Z-VAD-FMK, the proteasome inhibitor MG132, and the autophagy-lysosome inhibitor NH_4_Cl. When these inhibitors did not affect cell activity (Supplementary Figure 1), we explored the ability of the inhibitors to reduce IRF7 protein levels. The results showed that after the enzyme caspase was inhibited, the 3C-induced reduction in IRF7 expression was significantly inhibited, but MG132 and NH_4_Cl had no obvious effect (Figure 7). The caspase family consists of many members, the most important of which are caspase 3, caspase 8, and caspase 9, which play an important role in the process of apoptosis (Lai et al., 2019). To explore which caspase enzyme is needed for the 3C protein to reduce the expression of IRF7, we treated the cells with Z-DEVD-FMK, Z-IETD-FMK, and Z-LEHD-FMK, inhibitors of caspase 3, caspase 8, and caspase 9, respectively. When the three inhibitors did not affect cell viability (Supplementary Figure 2), we explored the effect of the three inhibitors on reducing IRF7 protein levels. The results showed that after caspase 3 was inhibited, the IRF7 protein level was significantly restored, but the inhibitors of caspase 8 and caspase 9 had no obvious effect (Figure 8).

### The 3C Protein Inhibits the Interferon Regulatory Factor 7 Protein to Promote DHAV-1 Replication

**FIGURE 9:**
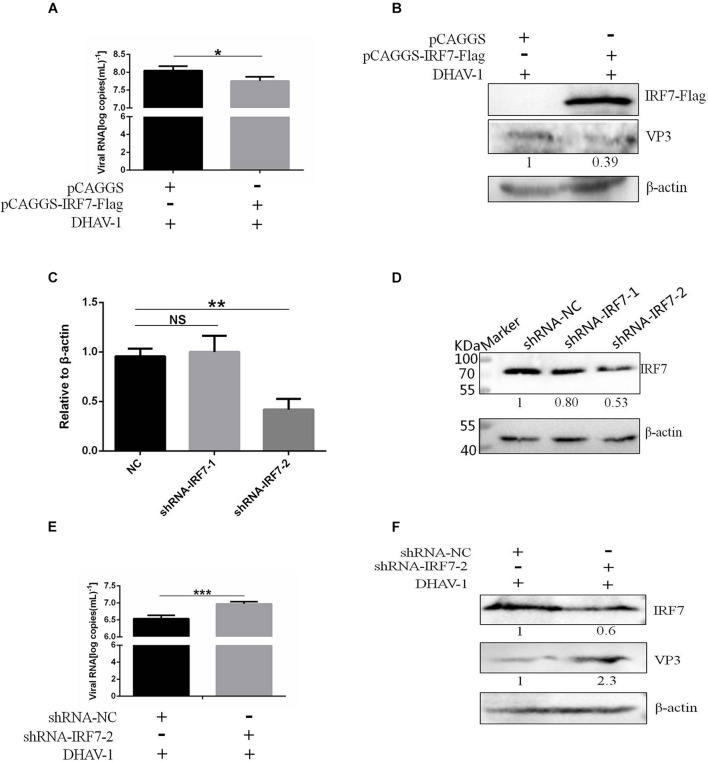
Effect of the IRF7 protein on DHAV-1 replication. pCAGGS-IRF7-Flag or shRNA-IRF7-1/2 was transfected into DEFs, and the pCAGGS and shRNA-NC negative control groups were set. Twenty-four hours after transfection, the cells were infected with the DHAV-1 CH strain at an MOI of 0.4, and cell samples were collected 36 h after transfection. **(A)** The viral copy number was detected when the IRF7 protein was overexpressed. **(B)** The structural protein VP3 was detected when the IRF7 protein was overexpressed. **(C)** The effects of shRNA-IRF7-1 and shRNA-IRF7-2 on the IRF7 mRNA level in DEFs. **(D)** The effects of shRNA-IRF7-1 and shRNA-IRF7-2 on IRF7 protein expression in DEFs. **(E)** The viral copy number was detected in DEFs transfected with shRNA-IRF7-2. **(F)** The structural protein VP3 was detected in DEFs transfected with shRNA-IRF7-2. *P < 0.05, **P < 0.01, ***P < 0.001, compared with the control group.

We proved that the 3C protein could induce the degradation of IRF7. As the most important transcription factor involved in type I IFN regulation in avian cells, the IRF7 protein plays an important role in the antiviral response of host cells. However, there have been no reports on the effect of the IRF7 protein on DHAV-1 replication. In this study, the effect of the IRF7 protein on DHAV-1 replication was evaluated. We transfected pCAGGS-IRF7-Flag and pCAGGS into DEFs. Compared with those in the pCAGGS transfection group, the viral copy number and VP3 protein expression levels in the pCAGGS-IRF7-Flag transfection group were significantly reduced (Figures 9A,B). Subsequently, we designed and synthesized two shRNAs named shRNA-IRF7-1 and shRNA-IRF7-2 based on the nucleic acid sequence of IRF7 and transfected them into DEFs. First, we evaluated the effects of the two shRNAs. Compared with shRNA-NC transfection, shRNA-IRF7-2 transfection significantly decreased the mRNA and protein levels of IRF7 (Figures 9C,D). After confirming that shRNA-IRF7-2 did not affect cell viability (Supplementary Figure 3), shRNA-IRF7-2 and the control plasmid, shRNA-NC, were transfected into DEFs. Compared with shRNA-NC transfection, shRNA-IRF7-2 transfection significantly increased the viral copy number and upregulated VP3 protein expression (Figures 9E,F), indicating that reduced IRF7 protein levels can promote DHAV-1 replication.

## Discussion

Like most viruses, for picornaviruses to successfully infect host cells, evading the host cell’s innate immune response is essential. The mechanisms by which picornaviruses such as EV71, PV, and CVB3 evade the innate immune response of host cells have been reported. Type I IFNs (IFN-α and IFN-β) are very important antiviral proteins of the host innate immune system. The key to inducing type I IFN is recognizing pathogen-associated molecular patterns (PAMPs) by pattern recognition receptors (PRRs). According to the literature, when picornaviruses invade cells, they are mainly recognized by the RLR receptor family, which includes RIG-I and MDA5, in host cells. Therefore, many picornaviruses suppress the innate immune response in host cells by inhibiting MDA5 or RIG-I. For example, PV disrupts the recognition system mediated by the MDA5 protein by lysing the MDA5 protein to successfully infect host cells (Barral et al., 2007). Furthermore, studies have found that the MDA5 protein is directly cleaved by the 2A protein (Feng et al., 2014). At the same time, a study found that the 2A proteins of both CVB3 and EV71 block the signaling pathway upstream of type I IFN by cleaving the MDA5 protein (Feng et al., 2014). Studies have also shown that the RIG-I protein is inhibited in EV71- and PV-infected cells. PV directly cleaves the RIG-I protein through its 3C protein (Barral et al., 2009; Papon et al., 2009). However, EV71 inhibits the formation of a complex between the RIG-1 and MAVS proteins through the interaction of its 3C protein and the RIG-I protein, thereby inhibiting the transcription of type I IFN (Lei et al., 2010). In addition, studies have shown that in cells lacking the RIG-I and MDA5 proteins, type I IFN, which is used to resist various RNA viruses, can be eliminated (Melchjorsen et al., 2005; Kato et al., 2006a,b; Takeuchi and Akira, 2009). Therefore, picornaviruses may be recognized by the RIG-I protein, the MDA5 protein, or both, thereby inducing type I IFN in various host cells.

In this study, we first used the dsRNA mimic poly(I:C) to stimulate the signaling pathway upstream of IFN-β in DEFs to screen for viral proteins in DHAV-1 that can regulate the signaling pathway upstream of IFN-β. The 3A, 3AB, and 3C proteins had a significant inhibitory effect on poly(I:C)-induced IFN-β promoter activation (Figure 1). The 3C proteins of other picornaviruses play an important role in regulating host immunity due to their proteolytic enzyme activity. Among the various viral proteins of DHAV-1, the 3C protein is the only protein with proteolytic enzyme activity confirmed thus far. We chose the 3C protein as the main research object in this study. The results showed that 3C protein inhibited poly(I:C)-induced IFN-β promoter activation in a dose-dependent manner and significantly reduced mRNA levels of the IFN-stimulating factors Mx, OASL, and IFN-β (Figure 2). The DHAV-1 3C protein was confirmed to have proteolytic enzyme activity, and histidine 38 and cysteine 144 are key sites for enzyme activity. After mutating these two amino acids to alanine, the DHAV-1 3C protein subsequently lost its proteolytic enzyme activity, indicating that these two amino acids are indispensable for its protease activity (Sun et al., 2017). When amino acid 38 in the 3C protein was mutated to alanine, poly(I:C)-induced IFN-β promoter activation was still significantly inhibited (Figure 2D), indicating that the inhibitory effect of the 3C protein on the signaling pathway upstream of IFN-β may not depend on 3C proteolytic enzyme activity. We transfected cells with eukaryotic expression plasmids to express the RIG-I N-terminal domain, MDA5 protein, MAVS protein, TBK1 protein, IRF7 protein, and the dual-luciferase reporter gene system was used to screen the target of the 3C protein, and we found that it inhibited the signaling pathway upstream of IFN-β. The 3C protein significantly inhibited activation of the IFN-β promoter induced by the RIG-I N-terminal domain, MDA5 protein, MAVS protein, TBK1 protein, and IRF7 protein (Figure 3), which indicated that the DHAV-1 3C protein mainly acts on the IRF7 protein and its downstream pathways to block the signaling pathway upstream of IFN-β.

Subsequently, we explored the effect of the 3C protein on the IRF7 protein. The nuclear and cytoplasmic separation results showed that the 3C protein prevented nuclear translocation of the IRF7 protein after poly(I:C) stimulation (Figure 4). These results indicate a specific interaction between the two proteins. We proved through coimmunoprecipitation experiments that the 3C protein indeed interacts with the IRF7 protein (Figure 6A) and binds the IRF7 protein through its N-terminus (Figure 6D), which may be related to the disruption of IRF7 nuclear translocation. However, the specific site where the IRF7 protein binds the 3C protein needs to be further explored.

For example, EV71 can directly cleave the IRF7 protein through its 3C protein, which targets glutamine 189 and serine 190 of the IRF7 protein to cleave it, thereby promoting the replication and proliferation of EV71 (Lei et al., 2013). Seneca Valley virus (SVV) is a new virus belonging to the family of small RNA viruses. Its 3C protein interacts with the IRF3 and IRF7 proteins in infected cells and degrades them to block the transcription of type I IFN. This process also depends on the proteolytic enzyme activity of the 3C protein (Xue et al., 2018). To explore whether the DHAV-1 3C protein directly cleaves it by interacting with the IRF7 protein, we expressed the 3C protein in DEFs and then detected the protein expression of IRF7. We did not detect a band indicating cleavage of the IRF7 protein, but the expression of the IRF7 protein decreased significantly with increasing 3C protein expression (Figure 5A). We mutated histidine 38 in the 3C protein to alanine, which caused the 3C protein to lose its proteolytic enzyme activity, but it could still reduce the protein expression of IRF7 (Figure 5B), also similar to the results in Figure 2D. These experimental results are consistent with those in Figure 2D. These results indicate that the 3C protein inhibits the signaling pathway upstream of IFN-β by inhibiting the IRF7 protein and that this process does not depend on its proteolytic enzyme activity. In the current literature, the immunoregulatory mechanisms related to the 3C protein of picornaviruses are all related to its proteolytic enzyme activity. Therefore, this study is the first to find that the 3C protein of a picornavirus regulates host cell immunity without relying on its proteolytic enzyme activity, which may reveal a new mechanism by which picornaviruses evade host immunity.

We have proven that the 3C protein affects the protein expression of IRF7 in a manner that does not depend on its proteolytic enzyme activity. Therefore, we speculated that the 3C protein indirectly decreases the protein expression of IRF7 by activating a protein degradation pathway in the host cells. In eukaryotic cells, proteins are usually degraded through three protein degradation pathways: ubiquitin-proteasome-dependent degradation, the autophagy-lysosomal pathway, and caspase-mediated protein degradation (Wang et al., 2017). We used the proteasome inhibitor MG132, autophagy lysosomal inhibitor NH_4_Cl, and broad-spectrum caspase inhibitor Z-VAD-FMK to treat cells. This experiment found that blocking the caspase-mediated proteolytic pathway restored IRF7 protein expression in cells expressing the DHAV-1 3C protein (Figure 7), indicating that the 3C protein degrades IRF7 protein in a caspase-dependent manner. Subsequently, we selected specific inhibitors of caspase 3, caspase 8, and caspase 9 to explore the mechanism of IRF7 protein degradation in more detail. Only the caspase 3 inhibitor prevented degradation of the IRF7 protein (Figure 8), which indicated that degradation of the IRF7 protein caused by the 3C protein is dependent on caspase 3. Previous studies found that DHAV-1 infection could activate apoptosis in cells (Lai et al., 2019). The activation of caspase 3 is crucial in the process of apoptosis (Buenz and Howe, 2006; Clarke and Tyler, 2009) and can mediate the hydrolysis of many host proteins in the final stage of apoptosis (Chi et al., 2013; Wang et al., 2017). EV71 cleaves the MDA5 protein in a caspase-dependent manner (Wang et al., 1998; Prévôt et al., 2003). It also degrades the nuclear localization signal receptor KPNA1 in a caspase 3-dependent manner, thereby preventing STAT1 in the pathway downstream of IFN-β from entering the nucleus (Wang et al., 2017). Its 2A protein can also degrade the IFN receptor IFNAR1 in a caspase 3-dependent manner (Chen et al., 2020). PV degrades the MDA5 protein in a proteasome- and caspase-dependent manner (Kuo et al., 2013). We discovered for the first time that the DHAV-1 3C protein mediates IRF7 protein degradation in a caspase 3-dependent manner rather than relying on its proteolytic enzyme activity.

Finally, we evaluated the effect of the IRF7 protein on DHAV-1 replication. In this experiment, we found that when we overexpressed the IRF7 protein, the viral copy number and expression of the structural protein VP3 were significantly reduced. When we reduced the protein expression of IRF7 in DEFs, the viral copy number and protein expression of VP3 was significantly increased (Figure 9), indicating that the antiviral protein IRF7 inhibits the replication of DHAV-1. These results also indicate that degradation of the IRF7 protein by 3C protein in a caspase 3-dependent manner may be a new mechanism to help DHAV-1 escape the IFN signal response in host cells, thereby promoting DHAV-1 replication.

In conclusion, we have demonstrated that the DHAV-1 3C protein inhibits nuclear translocation of the IRF7 protein and interacts with the IRF7 protein through its N-terminus. It also degrades the IRF7 protein through the caspase 3-dependent protein degradation pathway, thereby inhibiting natural IFN-β-mediated antiviral immunity and ultimately promoting the replication of DHAV-1. However, the specific mechanisms by which the 3C protein activates caspase 3 and uses caspase 3 to degrade the IRF7 protein need further exploration.

## Data Availability Statement

The datasets presented in this study can be found in online repositories. The names of the repository/repositories and accession number(s) can be found in the article/Supplementary Material.

## Author Contributions

YLL and XX carried out the experiments. AC and MW conceived of and supervised the study. MW, XO, SM, DS, SZ, QY, YW, RJ, DZ, SC, and ML contributed to this article’s conception. X-XZ, JH, QG, and BT helped draw the images. MW modified the manuscript. All authors reviewed the manuscript.

## Conflict of Interest

The authors declare that the research was conducted in the absence of any commercial or financial relationships that could be construed as a potential conflict of interest.

## Publisher’s Note

All claims expressed in this article are solely those of the authors and do not necessarily represent those of their affiliated organizations, or those of the publisher, the editors and the reviewers. Any product that may be evaluated in this article, or claim that may be made by its manufacturer, is not guaranteed or endorsed by the publisher.
