# Supplementary material for: DHAV-1 Blocks the Signaling Pathway Upstream of Type I Interferon by Inhibiting the Interferon Regulatory Factor 7 Protein
Source: Front Microbiol. 2021 Nov 12;12:700434. doi: 10.3389/fmicb.2021.700434 (PMC8633874; doi:10.3389/fmicb.2021.700434)
Supplement: Supplementary file 1 [file Data_Sheet_1.PDF]

Supplementary Material

1 Supplementary Figures

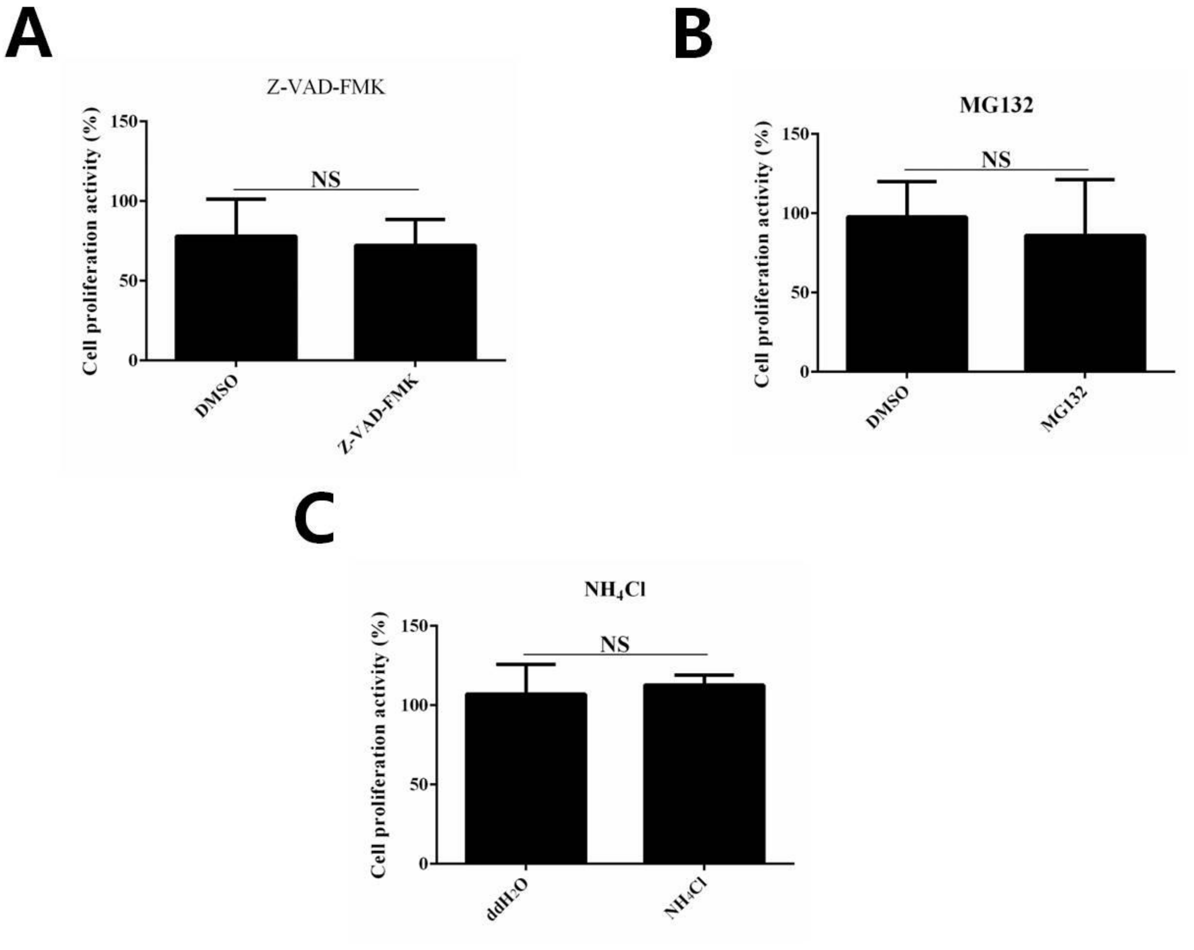

Supplementary Figure 1. The effect of various cell inhibitors on cell proliferation activity. (A) The effect of inhibitor Z-VAD-FMK on cell activity; (B) The effect of inhibitor MG132 on cell activity; (C) The effect of inhibitor NH_4Cl on cell activity.

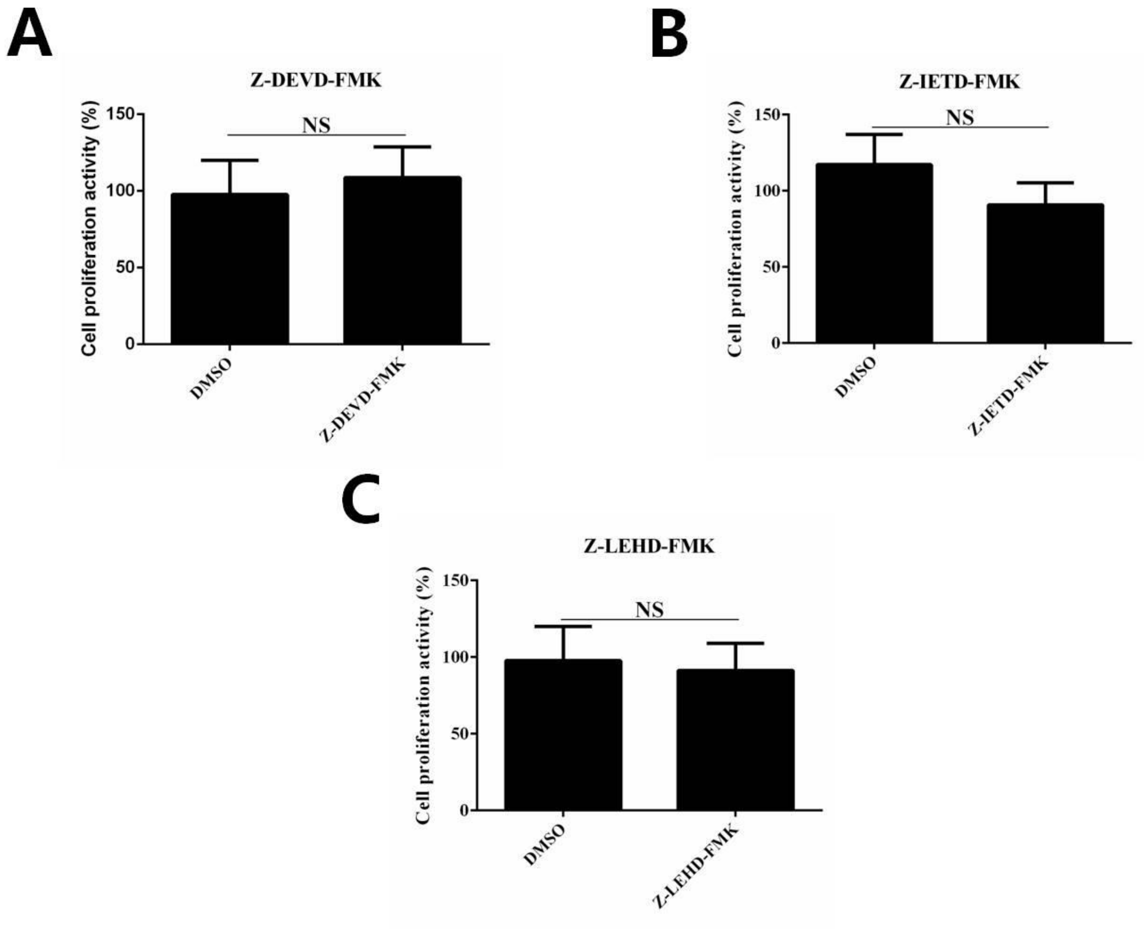

Supplementary Figure 2. The effect of various cell inhibitors on cell proliferation activity. (A) The effect of inhibitor Z-DEVD-FMK on cell activity; (B) The effect of inhibitor Z-IETD-FMK on cell activity; (C) The effect of inhibitor Z-LEHD-FMK on cell activity.

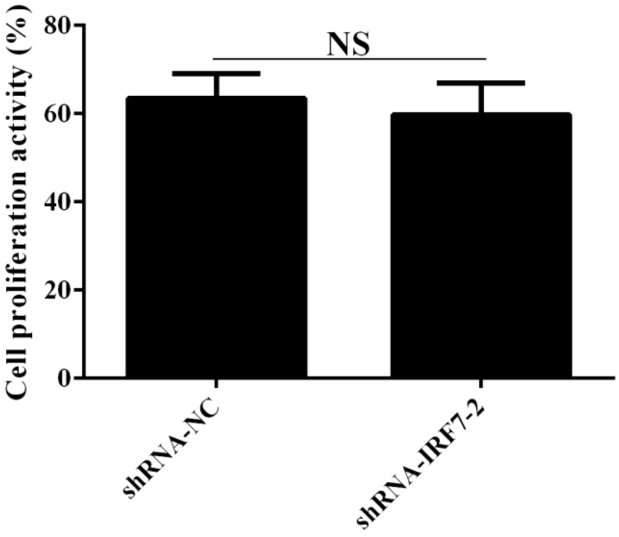

Supplementary Figure 3. The effect of shRNA-NC and shRNA-IRF7-2 on cell viability.
